# New Trends in Migraine Pharmacology: Targeting Calcitonin Gene–Related Peptide (CGRP) With Monoclonal Antibodies

**DOI:** 10.3389/fphar.2019.00363

**Published:** 2019-04-09

**Authors:** Damiana Scuteri, Annagrazia Adornetto, Laura Rombolà, Maria Diana Naturale, Luigi Antonio Morrone, Giacinto Bagetta, Paolo Tonin, Maria Tiziana Corasaniti

**Affiliations:** ^1^Preclinical and Translational Pharmacology, Department of Pharmacy, Health Science and Nutrition, University of Calabria, Cosenza, Italy; ^2^Department of Health Sciences, University “Magna Graecia” of Catanzaro, Catanzaro, Italy; ^3^School of Hospital Pharmacy, University “Magna Graecia” of Catanzaro, Catanzaro, Italy; ^4^Regional Center for Serious Brain Injuries, S. Anna Institute, Crotone, Italy

**Keywords:** migraine, pharmacology of migraine, CGRP, treatment, anti-CGRP, monoclonal antibodies anti-CGRP

## Abstract

Migraine is a common neurologic disorder characterized by attacks consisting of unilateral, throbbing headache accompanied by photophobia, phonophobia, and nausea which remarkably reduces the patients’ quality of life. Not migraine-specific non-steroidal anti-inflammatory drugs (NSAIDs) are effective in patients affected by mild episodic migraine whilst in moderate or severe episodic migraine and in chronic migraineurs triptans and preventative therapies are needed. Since these treatments are endowed with serious side effects and have limited effectiveness new pharmacological approaches have been investigated. The demonstrated pivotal role of calcitonin gene-related peptide (CGRP) has fostered the development of CGRP antagonists, unfortunately endowed with liver toxicity, and monoclonal antibodies (mAbs) toward circulating CGRP released during migraine attack or targeting its receptor. Currently, four mAbs, eptinezumab, fremanezumab, galcanezumab for CGRP and erenumab for CGRP canonical receptor, have been studied in clinical trials for episodic and chronic migraine. Apart from the proven effectiveness, these antibodies have resulted well tolerated and could improve the compliance of the patients due to their long half-lives allowing less frequent administrations. This study aims at investigating the still poorly clear pathogenesis of migraine and the potential role of anti-CGRP mAbs in the scenario of prophylaxis of migraine.

## Introduction: Migraine Clinic

Migraine consists in unilateral headache accompanied by a cluster of other sensory, autonomic and cognitive symptoms and it has been identified by the global burden of disease (GBD) study 2016 as the sixth most prevalent disorder and one of the main causes of disability all over the world, often occuring in working age and in young adult and middle-aged women (Collaborators, 2018). Hence, migraine represents a very serious social issue in terms of years of life lived with disability (YLDs) and the most important cause of YLDs within 15 to 49 years of age (see Steiner et al., 2018). The burden of such disease has not been identified until recently because: migraine is not a cause of permanent disability or death; headache occasionally occurs in general population (Collaborators, 2018). Although a definite pathogenesis for migraine is not known, the extracranial circulation is involved (Drummond and Lance, 1983). The clinical course of migraine is articulated in different following or concomitant stages: premonitory, aura, headache, and postdrome (similar to the premonitory phase) (Goadsby et al., 2017a). The premonitory phase includes irritability, food craving, stiff neck and can occur from 2 to 72 h prior to the attack and continues over the other phases. According to the definitions of the International Classification of Headache Disorders third edition (ICHD-3):

- aura is characterized by one or more transient, reversible neurological deficits, of which at least one has to have a unilateral localization, that develop over 5 min or more and of which each deficit lasts between 5 and 60 min;
- migraine consists in headache attacks lasting 4–72 h accompanied by nausea, photophobia and phonophobia, or both (see Goadsby et al., 2017a).

Cutaneous allodynia, defined as the perception of pain following non-painful stimuli, occurs in more than the 70% of patients (Lambru et al., 2018). Severe headache attenuates to stop during the postdrome phase, while other symptoms such as asthenia, somnolence and photophobia keep affecting the patient, thus displaying the complex neural basis underlying migraine (Lambru et al., 2018). Depending on the days per month affected, migraine is classified as episodic (fewer than 15 migraine or headache days) or chronic (at least 15 days, among which 8 or more are migraine days) (see Goadsby et al., 2017b). This aspect influences remarkably the impact of the disease and the therapeutic options (Giamberardino et al., 2017). With 1 to 3 attacks per month it is possible to use only abortive symptomatic drugs, while if 4 to 14 attacks per month occur it is mandatory to add preventative treatments; the latter treatments are needed to avoid chronification of the disease (Giamberardino and Martelletti, 2015; Giamberardino et al., 2017) and to reduce the risk of medication overuse headache and refractory migraine (Martelletti, 2017).

## Pathophysiology of Migraine and Calcitonin Gene–Related Peptide (CGRP)

**FIGURE 1:**
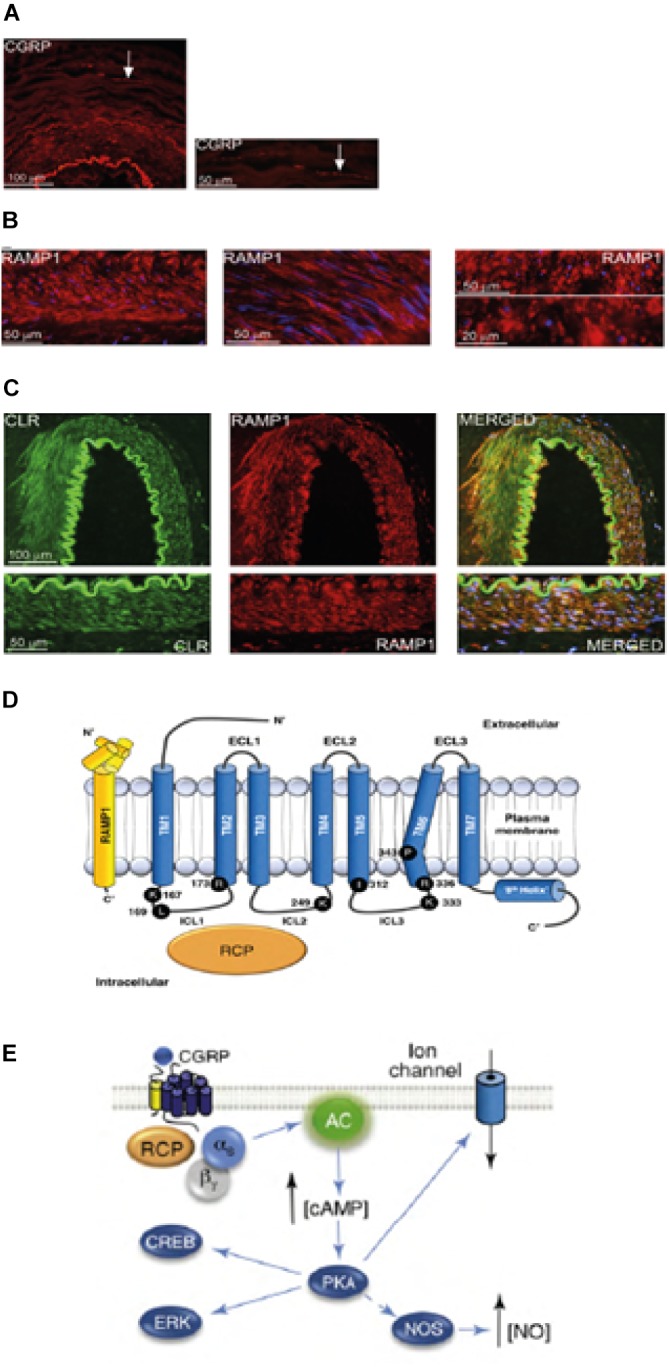
CGRP occurrence and pathway in migraine. Expression of CGRP, CLR, and RAMP1 in human dura vessels: **(A)** CGRP immunoreactivity is found in thin fibers in the adventitia; **(B)** cross and longitudinal sections showing RAMP1 expression in the cytoplasm of smooth muscle cells; **(C)** coexpression of CLR and RAMP1 in the smooth muscle cell layer. **(D)** CGRP receptor components and important residues for receptor signaling and internalization. The CGRP receptor is formed by CLR (blue), RAMP1 (yellow), and RCP (orange). Functionally important residues are shown as single letter abbreviations. Amino acid residues are numbered from the start of the predicted N-terminal signal peptide (Swiss-Prot Q16602). Several amino acids within the CLR C-terminus (∼N400-C436) and I312 at the ICL3/TM5 junction are required for effective CGRP-mediated internalization. Important features of the CGRP receptor, including the TM6 “kink” (P343) and the putative eighth helix (∼G388-W399) in CLR are illustrated. C′, C-terminal; ECL, extracellular loop; ICL, intracellular loop; N′, N-terminal; TM, transmembrane. **(E)** CGRP receptor-mediated intracellular signaling: Gα_s_ signaling increases AC (green) activity, elevating intracellular cAMP, activating PKA and subsequently many potential downstream effectors. Panels **(A–C)** are adapted from Eftekhari et al. (2013) and panel **(D,E)** from Walker et al. (2010), respectively, with permission.

The exact cause of migraine attacks is not well known yet, but the current research has been highlighting the importance of sensitization processes within the trigeminovascular system and the whole brainstem, as well as the observation of reduced gray matter in pain processing areas (see Goadsby et al., 2017a). From the trigeminal ganglion containing the cell body pseudo-unipolar primary afferents synapse on the blood vessels and on the trigeminocervical complex from which second-order fibers synapse on third-order thalamocortical neurons and on *locus coeruleus*, periaqueductal gray and hypothalamus (Goadsby et al., 2017a). The nociceptive fibers from the trigeminal ganglion and the cervical dorsal root ganglia innervate the dura mater vessels and their terminals release vasoactive neuropeptides such as calcitonin gene–related peptide (CGRP) inducing vasodilation. Expression of CGRP, CLR, and RAMP1 in human dura vessels is shown in Figure 1A–C. Indeed, the autonomic nervous fibers innervating extracerebral vasculature contain several neurotransmitter vasoactive molecules: noradrenaline, serotonin, acetylcholine, neuropeptide Y (NPY), vasoactive intestinal polypeptide (VIP), substance P (SP), neurokinin A (NU), and CGRP (Goadsby et al., 1990). The CGRP receptor antagonists act blocking the nociceptive information from the dura mater: it is processed in the thalamic nuclei from which it reachs higher cortical regions. These inputs from the trigeminovascular system are subjected to modulation by the brainstem descending pathway: the 5-hydroxytryptamine (5HT)1B/1D receptor agonists, i.e., triptans, are thought to act via this system and to counteract the occurred vasodilation. While propranolol and topiramate as preventive treatment, the latter drugs are used in acute episodes of migraine. In particular, apart from their vasoconstrictor activity, triptans directly acting on 5HT1B/D presynaptic receptors inhibit the release of mediators like CGRP involved in nociception. Furthermore, sumatriptan has experimentally been demonstrated to inhibit the inward current mediated by transient receptor potential vanilloid 1 (TRPV1) in the trigeminal ganglia (Evans et al., 2012). CGRP is released during migraine attacks and it displays several roles as the most vasoactive neuropeptide whose craniovascular levels increase in the course of the disorder (Goadsby et al., 1990). CGRP has been shown to undergo alterations also in cerebrospinal fluid (van Dongen et al., 2017). The triggers to altered central excitability and the following breakthrough of migraine pain episodes have long been investigated. The stimulation of nerve fibers can foster both orthodromic and antidromic action potentials and, in particular, the activation of dural peptidergic primary sensory afferents that express the transient receptor potential (TRP) can induce the release of several molecules including CGRP which trigger inflammatory tissue reactions known as neurogenic inflammation (Xanthos and Sandkuhler, 2014). In particular, CGRP is involved in cranial nociception and in the epiphenomenon of vasodilation binding its receptors on meningeal and cerebral blood vessels (Deen et al., 2017). These processes promote the sensitization of the trigeminal second order fibers taking amplified painful stimuli to higher regions like thalamus, hypothalamus and cortex, thus originating migraine (Dussor et al., 2014). TRPs are subjected to activation in response to several environmental irritant stimuli as temperature and pH variations that in predisposed individuals can trigger migraine pain. CGRP is produced by tissue-specific alternative splicing of the calcitonin gene CALC I on chromosome 11, also encoding for calcitonin. On the contrary, β CGRP is produced from CALC II gene located on a different site of chromosome 11. α CGRP neuropeptide is present in the central nervous system as α isoform of 37 amino acids and its signal transduction is mediated by two receptors.

The first one is known as canonical CGRP receptor and is a Gαs protein coupled receptor which requires the receptor activity-modifying protein (RAMP) 1 to be functional (Goadsby et al., 2017a). The second one is the human amylin subtype 1 receptor (AMY1): amylin belongs to the calcitonin gene family and it has hormonal activity. When CGRP binds to its receptor, it is subjected to dynamin/clathrin dependent internalization after having been complexed with β-arrestin (see Walker et al., 2010). Conformational changes occur inducing activation of adenylate cyclase (AC), increase in cAMP and activation of protein kinase A (PKA) that can promote vasodilation through direct activation of endothelial nitric oxide synthase and pain responses. This latter receptor can be coupled to Gαq/11 with the activation of phospholipase C (PLC), to mitogen-activated protein kinase (MAPK) and to the release of NO (see Walker et al., 2010) (see Figure 1D,E for schematic representation of CGRP receptor-mediated intracellular signaling). The administration of CGRP to migraineurs triggers attacks and this evidence supports its pivotal role in the pathogenesis of migraine (Lassen et al., 2002). A signal termination system has not been identified yet: CGRP is metabolyzed by neprilysin, insulin-degrading enzyme and endothelin-converting enzyme-1 and a reuptake active transport system has been hypothesized (see Russell et al., 2014). Moreover, when the receptor is transiently activated by CGRP it is internalized into endosomes and fastly recycled back to the cell membrane, while chronic stimulation of the receptor induces desensitization and lysosomal degradation (see Russell et al., 2014).

## Pharmacological Interventions on CGRP Pathway

Migraine is a multifaceted disabling neurovascular disorder and the current therapy with oral triptans is effective in acute attacks, though some 40% patients are resistant to treatment (Ferrari et al., 2001; Edvinsson, 2015). These selective 5-HT1B/1D agonists, of which the most commonly used is sumatriptan mostly active for subcutaneous route, exert their therapeutic action through vasoconstriction of cranial vessels, and inhibition of the trigeminal and trigeminocervical system (see Ferrari et al., 2001). However, vasoconstriction represents a limit for the cardiovascular side effects of these drugs. Furthermore, the treatment with triptans cannot last longer than 9 days per month because of the risk of drug-induced headache. In 2010 botulinum toxin type A (BoNT-A) was approved by the Food and Drug Administration (FDA) in the prevention of migraine in unresponsive chronic migraineurs and its complex administration (it requires injection in 31 sites) limits the compliance of patients. Due to the pivotal role played by CGRP in the pathophysiology of migraine, antagonists of its receptor have been developed. These drugs belong to the class of gepants. Telgecepant, the first oral gepant, resulted well tolerated in migraineurs affected by coronary artery disease (ClinicalTrials.gov NCT00662818, Ho et al., 2012), but it presented hepatotoxicity, thus, in spite of continuous research on these small molecules, a new approach consisting in the development of monoclonal antibodies (mAbs) toward CGRP (nezumabs) or CGRP receptor (numabs) was proposed. These drugs are believed to inhibit the action of the circulating CGRP molecules to prevent migraine attacks and, since they do not cross blood brain barrier (BBB) because of their size, the sites of action should be in the trigeminal system (Edvinsson, 2015). In particular, dural vessels are not included in BBB. Moreover, CGRP likely does not cross BBB and acts as a paracrine modulator since it is expressed by half neurons of trigeminal ganglion which do not present the CGRP receptor, expressed by satellite glial cells and the 30% of neuronal soma of trigeminal ganglion (see Yuan et al., 2017). Currently, there are four mAbs, eptinezumab, fremanezumab, galcanezumab and erenumab studied in clinical trials for episodic and chronic migraine. The first three mAbs are humanized antibodies directed toward CGRP, while erenumab is a human antibody toward its canonical receptor. The half-life of these drugs is quite long, thus allowing no more than one administration per month and this is a matter of interest since mAbs need to be administered for intravenous or subcutaneous route. In particular, eptinezumab only is administered intravenously (Israel et al., 2018). The phase III PROMISE 1 (PRevention Of Migraine via Intravenous eptinezumab Safety and Efficacy 1) trial (ClinicalTrials.gov NCT02559895) assessed Eptinezumab effectiveness on prevention of frequent episodic migraine. The results showed a significant reduction in the primary endpoint consisting in decrease of monthly migraine days (MMDs) from mean baseline of 8.5 days over weeks 1–12 to 4.3 MMDs with the dose of 300 mg, 3.9 with 100 mg, and 4.0 with 30 mg respect to 3.2 days of placebo (Saper et al., 2018). For the evaluation of eptinezumab in the prevention of chronic migraine 1121 participants have been enrolled in the PROMISE 2 (ClinicalTrials.gov NCT02974153). Galcanezumab was assessed for efficacy in the prevention of episodic migraine in the EVOLVE-1 phase 3 double-blind, randomized, placebo-controlled study (ClinicalTrials.gov NCT02614183) dissected in 4 periods and in the EVOLVE-2 Phase 3 randomized controlled double-blind 6 month-clinical trial (ClinicalTrials.gov NCT02614196). In the EVOLVE-1 both doses of galcanezumab (120 and 240 mg) met the primary outcome of significant reduction of monthly migraine headache days of 4.7 days and 4.6 days, respectively, in comparison with the 2.8 days of the placebo (Stauffer et al., 2018). In the EVOLVE-2 patients received monthly injection of 120 or 240 mg of galcanezumab. The mean monthly migraine headache days were reduced of 4.3 and 4.2 days, respectively, compared to 2.3 days reduction obtained with placebo (*p* < 0.001) (Skljarevski et al., 2018). Also the secondary endpoints of reduction of functional impairment assessed through the Role Function-Restrictive (R-FR) domain score of the Migraine-Specific Quality of Life Questionnaire (MSQ) and improvement of the score of the Patient Global Impression of Severity (PGI-S) and of the Migraine Disability Assessment (MIDAS; time point = month 6) were met (Skljarevski et al., 2018). 147 (65.0%) and 163 (71.5%) of the patients treated with galcanezumab, 120 and 240 mg, respectively, and 287 (62.3%) with placebo presented adverse events, among which acute myocardial infarction and transient ischemic attack within a group of seven patients under treatment with galcanezumab 240 mg (Skljarevski et al., 2018). There were not statistically significant differences in mean change from baseline for systolic/diastolic blood pressure. The 19 (8.6%), 11 (5.1%), and 2 (0.5%) patients in the galcanezumab 120 mg, galcanezumab 240 mg, and placebo groups, respectively, showed treatment-emergent anti-drug antibodies (ADA). In the REGAIN phase III trial evaluating galcanezumab against chronic migraine MMDs were reduced of 4.8 days with 120 mg and of 4.6 days with 240 mg dose compared to 2.7 of placebo (see Yuan et al., 2017). Moreover, the treatment with galcanezumab was demonstrated to be endowed with statistically significant persistence of the effect (Forderreuther et al., 2018). Fremanezumab was investigated in the phase III HALO trial for the preventive treatment of migraine (ClinicalTrials.gov NCT02638103). It reduced MMDs of episodic migraine at 12 weeks of 3.7 days at 225 mg monthly for 3 months and of 3.4 days at 675 mg once in quarterly dose regimen when compared to the 2.2 days decrease of placebo (see Yuan et al., 2017). The effectiveness of erenumab in episodic migraine prevention was studied in the phase 3, randomized, double-blind, placebo-controlled study ARISE (ClinicalTrials.gov NCT02483585). MMDs were reduced by 70 mg monthly subcutaneous erenumab of 2.9 days in comparison with 1.8 days of placebo (*p* < 0.001) and it was effective in the secondary endpoints of at least a 50% reduction in MMDs and change in monthly migraine-specific medication treatment days (MSMD) (Dodick et al., 2018). The proportion of patients presenting adverse events was similar between the group treated with the mAb and the placebo; 4.3% tested positive for anti-erenumab–binding antibodies through week 12, one of whom transiently positive for neutralizing antibodies but only at week 4 (Dodick et al., 2018). Also the phase III STRIVE clinical trial (ClinicalTrials.gov NCT02456740) assessed the effectiveness of erenumab for the prevention of episodic migraine. Erenumab was administered at 70 or 140 mg monthly for 6 months (Goadsby et al., 2017b). The baseline mean number of MMDs was 8.3 and the 70 mg and 140 mg doses reduced it of 3.2 and 3.7, respectively, compared to 1.8 days of placebo (with *p* < 0.001 for each dose vs. placebo) (Goadsby et al., 2017b). Also over the final 3 months of treatment each dose of erenumab fulfilled the secondary endpoints of at least a 50% reduction from baseline in the mean number of migraine days per month and reduction from baseline in both the Migraine Physical Function Impact Diary (MPFID) everyday-activities (MPFID-EA) and physical-impairment (MPFID-PI) (Goadsby et al., 2017b). In those patients with anti-erenumab antibodies only one in the group treated with 70 mg tested positive for neutralizing antibodies and the mAb was overall well tolerated in terms of creatinine levels, liver toxicity, total neutrophil counts and electrocardiographic function (Goadsby et al., 2017b). According to the authors one of the limits of this trial is that patients that had not shown therapeutic response to more than two classes of migraine-preventive drugs were excluded (Goadsby et al., 2017b). Furthermore, there are clinical trials investigation the effectiveness of fremanezumab (NCT02945046 and NCT02964338) and galcanezumab (NCT 02397473 and NCT02438826) in the prevention cluster headache (see Israel et al., 2018), that is primary headache characterized by severe unilateral pain in the periorbital area accompanied by tearing, conjunctival redness, and rhinorrhea (Vollesen et al., 2018). Indeed, it was demonstrated that continuous intravenous infusion through infusion pump of 1.5 μg/min of CGRP for 20 min (in 2 days separated by at least 7 days) to patients with episodic (active or remission phase) or chronic cluster headache elicited cluster-like attacks in patients in active phase or with chronic cluster headache, but not in remission phase (ClinicalTrials.gov NCT02466334) (Vollesen et al., 2018). The main PK advantages of mAbs are represented by long elimination half-life thus limiting the need for daily dosing and clearance by proteolysis. Due to the effects of gepants on liver and because of the vasodilatory properties of CGRP, the most feared risks of the inhibition of CGRP signaling consist in the hypothesized hepatotoxicity and cardiovascular theoretical risk (Yuan et al., 2017). The European Headache Federation (EHF) produced guidelines on the use of anti-CGRP mAbs applying to the Grading of Recommendation, Assessment, Development and Evaluation (GRADE) method and, when not possible, relying on the opinion of a panel of experts (Sacco et al., 2019).

## Pharmacokinetics (PK)/Pharmacodynamics (PD) Relationship of Anti-Migraine mAbs

The development of mAbs has represented a completely new approach to inhibit the CGRP pathway. The main PD improvement achieved using mAbs instead of small molecules is that it is easier to target the broad CGRP receptor–ligand-binding site (Taylor, 2018). The main PK advantages of mAbs are represented by long elimination half-life without need for daily dosing and clearance by proteolysis and the most feared risks of the inhibition of CGRP signaling consist in the hypothesized hepato-toxicity, due to the effects of gepants on liver, and cardiovascular theoretical risk, because of the vasodilatory properties of CGRP. However, following the journey of these mAbs from their production to the target, the first hindrance that these molecules meet is their administration because of their scarce oral bioavailability. Therefore, a parenteral route of administration is required and, in order to favor adherence to the treatment, half-life needs to be long (Taylor, 2018). Immunoglobulins (Ig)G1, 2, or 4 are the possibilities (Wang et al., 2008; Taylor, 2018). Apart from the origin of the IgG, eventual cross-reactivity and individual modifications of catabolism can affect half-life (Bonilla, 2008; Taylor, 2018). The technology used to produce mAbs relies on the use of hybridomas composed of cells in continuous division which produce determined clones of a single type of antibody, with no or low variability (Taylor, 2018). Based on the origin of the amino acids composing the mAb, it is possible to distinguish among chimeric (rodent immunization; maintenance of rodent Fragment antigen-binding region Fab, but introduction of human Fragment crystallizable region Fc), humanized (zumabs, with mouse Complementarity Determining Regions, CDRs, grafted to human Fab regions) and human (introduction of the whole sequence of the human antibody gene in the mouse that becomes humanized, as for instance the XenoMouse) mAbs (see Taylor, 2018). The CDRs influence the fitting to the target. Among the 4 mAbs anti-migraine only erenumab is human, while the grafted CDRs are from mouse for fremanezumab, rabbit for eptinezumab and likely mouse for galcanezumab (see Taylor, 2018). In particular, the only anti-CGRP receptor mAb is erenumab, which is a human IgG2λ, and the other three mAbs directed against sites of CGRP are: eptinezumab, a genetically engineered humanized IgG1k; fremanezumab, a humanized IgG2k; galcanezumab, a humanized IgG4 (Edvinsson et al., 2018). Erenumab (Tmax 3–14 days) requires monthly subcutaneous administration, as well as fremanezumab (Tmax 3–20 days) and galcanezumab (Tmax 7–14 days) (Taylor, 2018). On the contrary, eptinezumab (Tmax 4.8 h) is intravenously administered once every 3 months; it associates more rapidly and dissociates more slowly than fremanezumab and galcanezumab. Since mAbs are large sized proteins, they cannot easily cross BBB. Some CGRP receptors are outside the BBB, thus allowing the action of anti-CGRP mAbs (Edvinsson, 2018). Before binding, the antibody enters vascular endothelial cells via pinocytosis. Large apparent distribution volumes could depend on the tissue and the related capacity of binding of the mAb (Lobo et al., 2004; Taylor, 2018). Erenumab is the most novel approach, since it is the only one to target a fusion protein of the extracellular domains of human G protein-coupled receptor calcitonin receptor-like receptor CALCRL (required in the receptors for CGRP and adrenomedullin) and RAMP1 including the CGRP binding pocket (Edvinsson et al., 2018). It has been demonstrated to competitively inhibit the binding of [^125^I]-CGRP to the human CGRP receptor in human neuroblastoma cells (SK-N-MC) with a Ki of 0.02 ± 0.01 nM (Shi et al., 2016). Erenumab exerted potent and full antagonism of CGRP-stimulated cAMP accumulation with an IC50 of 2.3 ± 0.9 nM in functional assays performed in SK-N-MC (Shi et al., 2016). Moreover, it resulted 5000-fold more selective for CGRP receptor showing no agonist/antagonist effect on other human calcitonin family receptors including adrenomedullin, calcitonin, and amylin receptors up to the highest concentration tested of 10 μM (Shi et al., 2016). Fremanezumab might exert its effect on different vessels; in fact, it causes concentration dependent inhibition of CGRP induced vasodilation in pre-contracted human cerebral, meningeal and peripheral abdominal arteries (Ohlsson et al., 2018). In addition, CGRP may act also on neurons and glial cells and on the glymphatic (lymphatic-like), the latter being implicated in the expression of aura (see Messlinger, 2018). The likely absence of metabolism by liver enzymes could avoid drug–drug interactions. Elimination occurs through renal proteolysis of amino acids. Although clinical trials report that these antibodies are overall well tolerated, apart from the highlighted injection site pain, there is concern with their immunogenicity through production of ADA (Taylor, 2018). During a double-blind, randomized, placebo-controlled clinical trial (ClinicalTrials.gov NCT 01337596) evaluating the treatment with different regimens of single and multiple doses of galcanezumab, 11 of treated patients (26%) presented low titers (1:10 – 1:80) of treatment emergent-ADA (in 3 patients the pre-existing antibodies increased in titer), without dose-response and detected effects on PK and PD (Monteith et al., 2017). The study of immunogenicity is essential because the presence of ADA can accelerate drug removal or, worse, foster end organ damage (Taylor, 2018). The main characteristics of anti-migraine mAbs are summarized in Table 1.

**Table 1:** Main characteristics of fremanezumab, eptinezumab, galcanezumab, and erenumab.

mAb	PK	PD	Outcome
Fremanezumab	Monthly subcutaneous administration (Tmax 3–20 days) (Taylor, 2018).	Humanized anti-CGRP IgG2 (Edvinsson et al., 2018).	HALO (NCT02638103). Decrease in MMDs of episodic migraine at 12 weeks of 3.7 days at 225 mg monthly for 3 months and of 3.4 days at 675 mg once in quarterly dose regimen, in comparison with the 2.2 days of placebo (see Yuan et al., 2017).
Eptinezumab	Intravenously administered once every 3 months (Tmax 4.8 h) (Taylor, 2018).	Humanized IgG1 toward CGRP (Edvinsson et al., 2018).	PROMISE 1 (NCT02559895): activity in prevention of frequent episodic migraine from mean baseline of 8.5 days over weeks 1–12 to 4.3 MMDs with the dose of 300 mg, 3.9 with 100 mg and 4.0 with 30 mg respect to 3.2 days of placebo (Saper et al., 2018).
Galcanezumab	Monthly subcutaneous administration (Tmax 7–14 days) (Taylor, 2018).	Humanized anti-CGRP IgG4 (Edvinsson et al., 2018).	EVOLVE-2 (NCT02614196): effectiveness in prevention of episodic migraine. 120 mg or 240 mg of galcanezumab reduced the mean monthly migraine headache days of 4.3 and 4.2 days, respectively, compared to 2.3 days with placebo (Skljarevski et al., 2018). REGAIN: chronic migraine. MMDs were reduced of 4.8 days with 120 mg and of 4.6 days with 240 mg dose compared to 2.7 of placebo (see Yuan et al., 2017).
Erenumab	Monthly subcutaneous administration (Tmax 3–14 days) (Taylor, 2018).	Anti-CGRP receptor human IgG2 (Edvinsson et al., 2018).	ARISE (NCT02483585): prevention of episodic migraine. Reduction in MMDs by 70 mg monthly subcutaneous erenumab of 2.9 days in comparison with 1.8 days of placebo (Dodick et al., 2018). STRIVE (NCT02456740): prevention of episodic migraine. 70 mg and 140 mg doses (monthly for 6 months) reduced baseline mean number of MMDs (8.3) of 3.2 and 3.7, respectively, compared to 1.8 days of placebo (Goadsby et al., 2017b).


*The administration route and frequency, structure of the mAb and target and the main results of the clinical trials of the four mAbs acting on CGRP-pathway are outlined.*

## Conclusion

Migraine is a disabling and debilitating neurovascular painful condition representing more than 90% of cases of recurrent headache and toward which the tendency can be inherited (MacGregor, 2017). Divalproex sodium, sodium valproate, topiramate, metoprolol, propranolol, and timolol have proven strong, level A, evidence for migraine prevention (American Academy of Neurology and American Headache Society, 2015). However, all the classic oral preventative treatments including tricyclic antidepressants, beta blockers, 5-HT2 antagonists ergots and anti-epileptic drugs were not developed for migraine and provide 50% reduction in the number of monthly days of migraine pain only up to the 45% of migraineurs (D’Amico and Tepper, 2008), also because of low adherence due to scarce tolerability. Medication persistence and discontinuation was examined in a retrospective US claims analysis (Hepp et al., 2017) and the results suggest low persistence to the initial drug used and high amount of discontinuation by 6 months independently on drug class. Due to the fundamental role of CGRP in sustaining neuroinflammation and central sensitization in the pathway from trigeminal ganglion and brainstem to higher regions involved in the physiopathology of migraine, novel mAbs toward CGRP and its receptor have been developed. The first small molecules CGRP antagonists showed to induce liver toxicity, but mAbs did not produce either toxic metabolites or cardiovascular side reactions because of the inhibition of vasodilation (see Deen et al., 2017). Central side effects were not highlighted, as well. The potential long-term effects of blocking CGRP still need to be studied but, a fundamental advantage of these antibodies stems from their long half-life allowing monthly or less frequent injections, which can remarkably improve adherence to the treatment and its following effectiveness (Deen et al., 2017). Thus, anti-CGRP mAbs could represent effective tools in the therapeutic arsenal against unresponsive migraine; however, deep monitoring of efficacy and safety (i.e., production of toxic metabolites, immunogenicity with ADA, neutralizing antibodies and tissue cross-reactivity and side effects) is mandatory (Taylor, 2018). In fact, evidence from older biotech products show that therapies with anti-tumor necrosis factor α (TNFα) mAbs can be subjected to secondary failure (in contrast with primary non-responders) of the initial therapeutic response because these drugs induce the production of antibodies that may degrade the mAb but may also neutralize its action before binding to the target (see Prado et al., 2017). Immunogenicity can be influenced by glycosylation, type of mAb, number of epitopes and impurities in formulation (see Prado et al., 2017). Moreover, several apparently unexplainable serious side effects are associated with immunogenicity of anti-TNFα. Local and systemic hypersensitivity reactions, immunodeficiency with increased susceptibility to infections, and immune complex formation which can lead even to death have been highlighted (see Prado et al., 2017). Therefore, the use of therapeutic diagnostics (theranostics) may improve the knowledge and correct management of these pharmacological devices (Bendtzen, 2013; Prado et al., 2017).

## Author Contributions

MTC, LAM, PT, and GB conceived the study. DS collected the trial results, analyzed the literature, and wrote the manuscript. AA, LR, and MN participated in the literature survey. All authors read and approved the final manuscript.

## Conflict of Interest Statement

The authors declare that the research was conducted in the absence of any commercial or financial relationships that could be construed as a potential conflict of interest.
